# Characterizing the extent human milk folate is buffered against maternal malnutrition and infection in drought‐stricken northern Kenya

**DOI:** 10.1002/ajpa.24603

**Published:** 2022-08-23

**Authors:** Masako Fujita, Katherine Wander, Tin Tran, Eleanor Brindle

**Affiliations:** ^1^ Department of Anthropology Michigan State University East Lansing Michigan USA; ^2^ Biomarker Laboratory for Anthropological Research Michigan State University East Lansing Michigan USA; ^3^ Department of Anthropology Binghamton University (SUNY) Binghamton New York USA; ^4^ Laboratory for Anthropometry and Biomarkers Binghamton University Binghamton New York USA; ^5^ College of Pharmacy University of Iowa Iowa City Iowa USA; ^6^ Maternal, Newborn and Child Health & Nutrition PATH Seattle Washington USA

**Keywords:** anemia, breastfeeding, chronic energy deficiency, inflammation, lactation, micronutrient deficiency

## Abstract

**Objectives:**

Folate is an essential nutrient fundamental to human growth and development. Human milk maintains high folate content across the maternal folate status range, suggesting buffering of milk folate with prioritized delivery to milk at the expense of maternal depletion. We investigated whether and how the extent of this buffering may diminish under prolonged nutritional and/or disease stress, while taking into consideration infants' varying vulnerability to malnutrition‐related morbidity/mortality.

**Methods:**

A cross‐sectional study analyzed milk specimens from northern Kenyan mothers (*n* = 203), surveyed during a historic drought and ensuing food shortage. Multiple regression models for folate receptor‐α (FOLR1) in milk were constructed. Predictors included maternal underweight (BMI < 18.5), iron‐deficiency anemia (hemoglobin <12 g/dl and dried‐blood‐spot transferrin receptor >5 mg/L), folate deficiency (hyperhomocysteinemia, homocysteine >12 or 14 μmol/L), inflammation (serum C‐reactive protein >5 mg/L), infant age and sex, and mother‐infant interactions.

**Results:**

In adjusted models, milk FOLR1 was unassociated with maternal underweight, iron‐deficiency anemia and inflammation. FOLR1 was positively associated with maternal folate deficiency, and inversely associated with infant age. There was interaction between infant age and maternal underweight, and between infant sex and maternal folate deficiency, predicting complex changes in FOLR1.

**Conclusions:**

Our results suggest that mothers buffer milk folate against their own nutritional stress even during a prolonged drought; however, the extent of this buffering may vary with infant age, and, among folate‐deficient mothers, with infant sex. Future research is needed to better understand this variability in maternal buffering of milk folate and how it relates to folate status in nursing infants.

## Research highlights

Human mothers might have the capacity to buffer milk nutrient content against undue fluctuations under nutritional or disease stress. In the case of the micronutrient folate, this buffering is most apparent for younger infants.

## 1 INTRODUCTION

Mothers' milk nourishes and protects infants by delivering essential nutrients, immune factors, and other bioactive compounds (Miller et al., 2013). The macronutrient and energy content in human milk, and more generally the milk of primates, are buffered against short‐term maternal nutritional stress (Miller et al., 2013; Prentice et al., 1981; 1994; Villalpando & del Prado, 1999). This buffering of milk nutrients is possible through a combination strategy to draw nutrients from both dietary sources (‘income’) and somatic nutrient reserves (‘capital’) for milk synthesis (Hinde et al., 2009). This strategy is not shared by other mammals, such as rodents, whose milk nutrients originate (Black, 2008) predominantly in their ‘income’ (Hinde et al., 2009).

Here, we expand on the maternal buffering hypothesis by evaluating whether human milk micronutrient content (folate) may be similarly buffered against maternal nutritional or disease stress. Using data from a harsh environment with prolonged drought, we characterize the pattern of variation in milk folate content to allow for an understanding of the extent of maternal buffering of milk folate against different forms of maternal nutritional and infectious disease stress. We integrate the maternal buffering with a complementary concept—the ‘protective’ hypothesis—that milk nutrient content increases in proportion to infant needs (e.g., needs for protection against malnutrition or infectious disease [Breakey et al., 2015; Fujita et al., 2019; Fujita, Paredes Ruvalcaba, Wander, et al., 2018]). To this end, we evaluate whether and how maternal and infant characteristics may interact to affect milk folate content.

### 1.1 Maternal buffering

The maternal buffering hypothesis asserts that evolution has shaped primate and human lactation to be capable of buffering milk nutritional value against maternal malnutrition (Miller et al., 2013; Prentice et al., 1981; 1984; 1994; Villalpando & del Prado, 1999). Buffering is possible through mobilization of maternal body reserves, which, while they last, can continue to nourish milk when dietary sources of nutrients dwindle (Hinde et al., 2009). Previous studies have provided some support for maternal buffering of milk energy content (Fujita, Paredes Ruvalcaba, Wander, et al., 2018; Lönnerdal, 1986; Mandel et al., 2005). Less is known about maternal buffering of milk micronutrient content.

Micronutrients in milk have been categorized into two groups based on the degree to which their concentrations vary in relation to maternal status and intake. Group I nutrients are those that tend to decrease when maternal status or intake decreases, and include vitamin B6, vitamin B12, and retinol, among others (Allen, 2012). Group II nutrients are more robust to maternal malnutrition or inadequate dietary intake. These include folate (Allen, 2012), which appears to maintain remarkably high concentrations. Researchers have interpreted these findings to mean that maternal physiology gives high priority to delivering folate to infants, even at the expense of maternal folate depletion (Allen, 2012; Metz, 1970; Salmenpera et al., 1986). Existing literature therefore characterizes milk folate as fairly invariant.

For anthropologists interested in human adaptability, this characterization of milk folate as invariant is a *hypothesis* worthy of additional scrutiny. Although milk folate content may be generally robust to the effects of maternal folate deficiency among the populations well‐represented in the literature, less is known about whether or how milk folate may vary across the full range of human variation, including contexts of severe and multifaceted ecological stress that can result in prolonged periods of poor nutrition among mothers. Although a stable supply of milk folate in such settings is important for infants, mothers also need folate for their immediate physiological needs, long‐term health, and future reproduction (Bailey & Gregory, 1999; Bartley et al., 2005; Jablonski & Chaplin, 2000; Tamura & Picciano, 2006). The maternal costs of buffering milk folate (i.e. less folate available for mothers themselves) are likely to be high and influenced by the extent and duration of maternal malnutrition. This leads us to expect that the extent of maternal buffering of milk folate should vary with maternal folate nutrition and other stressors.

### 1.2 Maternal protection

The protective framework views milk content as maternal effort to protect the infant against nutrient shortfalls and/or infectious diseases, and therefore, expects maternal delivery of milk content to vary with differing infant needs (Breakey et al., 2015; Fujita et al., 2019). From this perspective, we expect mothers to increase delivery of milk nutrients or protective factors as infants' risk for mortality from malnutrition and/or infectious diseases increases (and conversely to decrease delivery as risk decreases).

### 1.3 Folate nutrition

Folate is an essential nutrient, utilized by nearly all organisms for multiple biological functions, including nucleic acid and protein biosynthesis (Gorelova et al., 2019). Folate is important for fetal and infant development and sustained growth (e.g., via methylation of DNA and histones on the genes that code for development [Tamura & Picciano, 2006]). Folate is also important for competent cell‐mediated immune responses (Courtemanche et al., 2004).

Given the fundamental biological function of folate, it is not surprising that fetal and infant demand for folate are elevated. Folate needs are correspondingly high for mothers during pregnancy and lactation (Jablonski & Chaplin, 2000). Folate deficiency among women at pre‐conception increases the risk of congenital defects and pregnancy failure (Bartley et al., 2005). For this reason, a synthetic form of folate, folic acid, is widely utilized for maternal supplementation during pregnancy to prevent neural tube defects in offspring (Tamura & Picciano, 2006).

Postpartum, breastfed infants obtain folate through milk. Compared to other dietary sources of folate (e.g. legumes, cereals, green vegetables), milk folate has superior bioavailability. Previous research has found that milk folate concentrations are maintained even among mothers with folate deficiency, and are unaffected by folic acid supplementation (Cooperman et al., 1982; Houghton et al., 2009; Mackey & Picciano, 1999; Trugo et al., 1988). Maintaining an adequate milk folate concentration to support infant development may come at the expense of maternal well‐being (O'Connor et al., 1997; Tamura & Picciano, 2006). However, low milk folate concentrations can occur when maternal folate levels are abnormally low (O'Connor et al., 1997) or in cases of experimentally induced folate deficiency (Metz, 1970).

### 1.4 Folate receptor‐α

In human milk, folates occur predominantly in a reduced form, 5‐methyl‐tetrahydrofolate (Tamura & Picciano, 2006), that is highly labile and prone to consumption by microflora (Nygren‐Babol & Jagerstad, 2012) and prone to breakdown by UV exposure (Lucock et al., 2017). The delivery of delicate milk folate to infants therefore relies on a folate binding protein called folate receptor‐α (FOLR1). FOLR1 is a soluble and glycosylated protein that forms a secure complex with folate. (Holm & Hansen, 2020; Nygren‐Babol & Jagerstad, 2012; Rosenberg & Selhub, 2006; Selhub et al., 1984).

The milk folate‐FOLR1 complex is a highly efficient means of folate transfer to infants that sets mothers' milk apart from other folate sources. In the mammary gland, FOLR1 proteins function to concentrate folate multifold from maternal plasma to milk (FOLR1 can be 10‐fold to 1000‐fold more concentrated in milk than blood plasma [Fujita, Paredes Ruvalcaba, & Corbitt, 2018; R&D, 2011; Tamura et al., 2009]; the magnitude of difference likely depends on the method of analysis; see [Nygren‐Babol & Jagerstad, 2012]). FOLR1 stabilizes folate, prevents enzymatic degradation, and increase folate bioavailability to infants (Ford, 1974; Mason & Selhub, 1988; Nygren‐Babol & Jagerstad, 2012). FOLR1 also prevents bacteria in the infant gut from accessing maternal folate, similar to lactoferrin sequestering iron from microorganisms (Trugo et al., 1988). In milk, folate and folate binding protein are highly positively correlated (*r* = 0.71) among US mothers (Selhub et al., 1984; Tamura et al., 2009). FOLR1 preserves well under cryogenic temperatures and tolerates multiple freeze–thaw cycles (Balion, 2011); the stability of FOLR1 makes it an excellent biomarker for milk folate content.

### 1.5 Milk folate content variation by maternal nutrition and inflammation

Consensus is currently lacking regarding the effects of different aspects of maternal nutrition (other than folate status), inflammation and infection on milk folate concentration. There is some evidence that maternal delivery of milk folate may be influenced by non‐folate maternal nutrition (Donangelo et al., 1989; Oconnor et al., 1987). A study in Brazil found milk folate content was positively associated with maternal serum vitamin B12, zinc, and albumin even though it was unassociated with maternal serum folate, iron, and ferritin (Donangelo et al., 1989). A study in Mexico found no difference in milk folate concentrations by maternal iron deficiency or low blood folate (Khambalia et al., 2006; Villalpando et al., 2003), contradicting prior research with animal models suggesting that milk folate secretion can decline during iron deficiency (Oconnor et al., 1987).

While we are aware of no studies that specifically investigated the effect of maternal inflammation or infection on milk folate content, the broader literature provides clues that inflammation or infection may influence milk folate. This includes the reported associations between some chronic inflammatory bowel conditions and low blood folate, likely due to low nutrient absorption (Stabler, 2010), and a positive association between serum C‐reactive protein (CRP, an acute phase reactant and biomarker of inflammation) and folate deficiency among lactating indigenous women in Panama (Gonzalez et al., 2017). Furthermore, our previous studies have linked elevated CRP among lactating mothers to altered nutrient content in their milk, including fat (Fujita, Paredes Ruvalcaba, Wander, et al., 2018) and retinol (Fujita et al., 2017) in Kenya, although observations in Malawi contradict the latter (Dancheck et al., 2005).

Lack of consensus also extends to the effect of infant characteristics on milk folate content. Studies report increases, decreases, or no change in milk folate contents in association with infant age (e.g., [Donangelo et al., 1989; Mackey & Picciano, 1999; Tamura et al., 2009]), with discrepancies likely due in part to differences in maternal folate status (Tamura et al., 2009). Clarifying the roles maternal nutritional and disease status play in milk folate content can help us better understand variation in postnatal folate transfer in ecological context, with important implications for infant growth and development.

### 1.6 Maternal buffering and protection combined: hypotheses

**FIGURE 1:**
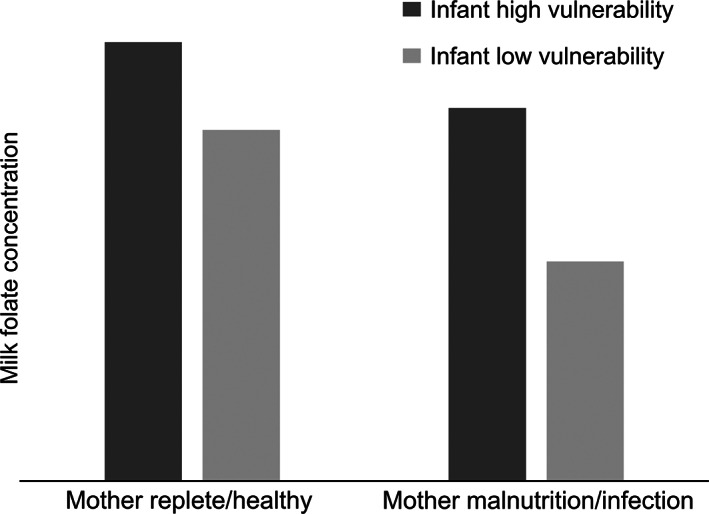
Hypothesized milk folate content variation patterns from the combined perspective of maternal buffering and protection

We expect milk folate delivery to be buffered against maternal nutritional or disease stress, but the extent of buffering to vary in ways reflecting maternal protective effort. Buffering milk folate entails high opportunity cost to undernourished mothers (i.e. less folate available to support their own health), and therefore we broadly predict that mothers with nutritional deficiencies or inflammation/infection will imperfectly buffer milk folate. We further broadly predict more protective effort (i.e. higher milk folate) for infants who are more vulnerable to physiological effects of folate shortfalls and have higher needs for maternal protection (e.g., younger infants undergoing rapid cellular processes requiring folate [Black, 2008]). Finally, we predict the difference by infant vulnerability to be more apparent among mothers with nutritional deficiencies or inflammation/infection (due to more robust buffering in effort to protect more vulnerable infants and attenuated buffering for less vulnerable infants) than mothers under less stress; Figure 1.

### 1.7 Study objectives

We conducted a secondary analysis of the cross‐sectional data from 203 predominantly normal‐to‐underweight, seemingly healthy breastfeeding mothers within 20 months postpartum, collected during drought‐induced food scarcity in northern Kenya. The study objectives were to evaluate whether milk folate delivery is buffered against maternal nutritional or disease stress; whether milk folate buffering is incomplete among some mothers; and whether milk folate delivery exhibits maternal protection (enhanced delivery to infants most vulnerable to ill effects of folate deficiency).

## 2 MATERIALS AND METHODS

The study assessed associations of milk folate with maternal nutritional status and elevated inflammation (a likely indicator of infectious disease), infant age and sex, and interactions between maternal and infant variables.

### 2.1 Study setting and specimen/data source

We utilized cryogenically archived milk specimens and associated data from seemingly healthy but marginally nourished breastfeeding mothers ≥18 years of age (*n* = 203). The specimens and data originated in a study among Ariaal agro‐pastoralists in three locations (Karare, Kituruni, and Korr) in Marsabit District of northern Kenya where malnutrition is prevalent and infectious morbidity and mortality are high (Kenya National Bureau of Statistics & ICF Macro, 2010; Miller, 2011). The ecological, historical, sociocultural and epidemiological contexts of Ariaal agro‐pastoralists have been described elsewhere; this population traditionally persisted in the remote, arid terrain of northern Kenya via nomadic pastoralism but at the time of data collection had settled in highland (Karare and Kiturni) or lowland (Korr) sedentary communities since the 1970s as participants in the agricultural development project on Mount Marsabit or for other opportunities (Fratkin, 1998; Fratkin et al., 2004; Fratkin & Roth, 1990; Fujita, 2003; Fujita et al., 2004; 2005; Shell‐Duncan & Yung, 2004).

The original data collection occurred during the 2006 Horn‐of‐Africa drought, one of the most serious droughts during the recent history of East Africa. The drought caused major loss of livestock and agricultural crops in northern Kenya. This intensified food insecurity and many residents were dependent on drought relief foods (Fujita, 2008). In addition to two consecutive years of failed rainfall (2004–2005), the drought was compounded by conditions leading into it, including poor livestock health, decimated herd sizes, and high food prices (Marsabit District, 2006;USAID, 2018; World Health Organization, 2006). The government response (famine food relief) was also slow, contributing to the severity of the problem (World Health Organization, 2006). As such, food insecurity had been chronically high for at least 2 years at the time of data collection.

Data collection took place from August through early September of 2006; fodder/livestock conditions had begun to improve in lowland areas at this time, as some precipitation had occurred. Household‐level food insecurity, regardless of the location, remained high throughout the data collection, with no notable improvement until November or December of 2006. Nutritional and health conditions were worst around the time of data collection (e.g., childhood diarrhea cases peaked in early August [World Health Organization, 2006]).

The surveyed mothers were 1–20 months postpartum, meaning all carried their pregnancies during the extended period of failed rainfall and food scarcity. The original study did not collect any data on gestation or birth weight. In the present study therefore we assume all pregnancies were similarly affected by the drought and famine. In reality, however, the impact of food and water scarcity likely differed between mothers, depending on the timing of pregnancy relative to the timing of socioecological events such as the onset of famine relief distributions (USAID, 2018; World Health Organization, 2006).

Data from the original and subsequent studies have revealed high rates of maternal malnutrition, anemia, and inflammation/infection (Fujita et al., 2011; 2012; 2014; Fujita & Wander, 2017; Paredes Ruvalcaba et al., 2020). For the present study, we drew on a convenience subsample of participants for whom all relevant data were complete, of the random sample of 241 mothers representing three communities in the original study. The study was approved and overseen by the institutional review boards of the University of Washington and Kenya Medical Research Institute. All mothers provided informed consent. The analysis of de‐identified milk specimens and data presented here required no further approvals.

#### 2.1.1 Milk FOLR1 concentration estimation

The milk utilized for the determination of milk FOLR1 were foremilk specimens expressed manually by mothers into a disposable cup (assisted by female staff, as needed). Mothers were asked to fast overnight and refrain from use of one breast for overnight feeding, for the original purpose of determining milk retinol concentration, as described elsewhere (Fujita, 2008; Fujita et al., 2011). Upon collection, the milk was immediately transferred to an opaque bottle to minimize UV exposure and frozen in liquid nitrogen. This sampling procedure (morning specimens of fasting milk from the breast not nursed overnight) allowed reducing the influence of immediate meals, breastfeeding, and possible diurnal patterns in milk synthesis on milk retinol in our original research, and on milk FOLR1 in the present study.

The specimens were maintained frozen at cryogenic temperatures, except for undergoing two freeze–thaw cycles. The aqueous fraction of milk specimens was obtained by triple‐centrifuging thawed and homogenized milk at 4°C, as described elsewhere (Fujita et al., 2019). Milk FOLR1 concentrations were estimated in 2017 in the Biomarker Laboratory for Anthropological Research at Michigan State University with an enzyme immunoassay kit validated for use in human milk (Quantikine ELISA Human FOLR1 Immunoassay DFLR10 [R&D, 2011]). The intra assay CVs were ≤7.1% and the inter‐assay CVs were ≤6.8% for the controls of low and high concentrations across seven plates.

#### 2.1.2 Maternal nutritional and inflammation/infection status

We used underweight, iron deficiency anemia, and hyperhomocysteinemia to characterize maternal nutrition, and elevated CRP to characterize maternal inflammation. All these data were available from our previous research. Maternal status variables were all dichotomous indicator variables (no = 0/yes = 1), including underweight, iron deficiency anemia, hyperhomocysteinemia, and inflammation/infection. BMI < 18.5 defined underweight. Low hemoglobin levels (<12 g/dl) (World Health Organization, 2017) co‐occurring with elevated soluble transferrin receptor (>5 mg/l measured in dried blood spots) defined iron deficiency anemia. Hemoglobin and transferrin receptor concentrations were based on a portable hemoglobinometer (Hemocue Hb201+) and an ELISA kit (TFC‐94 Ramco Laboratories), respectively, as described in (Fujita & Wander, 2017; Wander et al., 2009). Hyperhomocysteinemia is a condition of elevated homocysteine (a sulfur amino acid) in blood. Homocysteine metabolism is catalyzed by enzymes that are dependent on folate, vitamin B12, and vitamin B6. Hyperhomocysteinemia is commonly utilized as a biomarker of folate deficiency, but occurs when any of these B‐vitamins are in short supply (McKay & Mathers, 2016). As such, it is a functional indicator of deficiency in folate, vitamin B6, and/or vitamin B12. There is no consensus on the cutoff point for hyperhomocysteinemia, with studies generally adopting a homocysteine value within the 10–20 μmol/L range, most commonly between 12 and 15 μmol/L (Aparicio‐Ugarriza et al., 2015). Hyperhomocysteinemia in this study therefore was defined using two alternative cutoffs >12 and >14 μmoL/L (following [Baily, 1998; Green, 2011], respectively) serum homocysteine estimated by an ELISA kit (Cat.# DE 2925, Demeditec Diagnostics GmbH [Tin & Fujita, 2020]). Elevated inflammation, likely due to acute subclinical infections (or injuries) rather than chronic disease processes in this normal‐to‐underweight population, was identified using elevated blood serum CRP (>5 mg/L; [Brindle et al., 2010; Freedman, 2001; Fujita & Wander, 2017; Nakagomi et al., 2000]).

#### 2.1.3 Infant age and sex

Infant age (in months) was determined from the infant's date of birth reported in a one‐on‐one interview with the mother. Infant sex (female/male) was also based on maternal report. Infant vulnerability to malnutrition and infectious disease was considered higher in infants of younger ages and male sex. We consider vulnerability here specifically with regard to physiological needs for folate; younger infants undergo more rapid cell divisions for growth and development than older infants (Black, 2008). As such, shortfalls in folate may have disproportionate ill effects on younger infants. This may be particularly notable in stressful environments. In northern Kenya (more broadly Eastern province of Kenya), infant mortality reported from 2000s is substantially higher among neonates than post‐neonates (Kenya National Bureau of Statistics & ICF Macro, 2010). Infants during the initial 3 months of life are more sensitive to growth faltering under a stressful environment compared to those in the subsequent 3 months (Madan et al., 2019). Male infants tend to be more susceptible to infection and growth faltering under stress (Naeye et al., 1971; Stinson, 1985) than female infants of the same age.

### 2.2 Statistical analysis

We fit a series of multiple regression models using natural log‐transformed milk FOLR1 as the dependent variable. Predictors included maternal underweight, iron deficiency anemia, hyperhomocysteinemia, inflammation, infant sex, and infant age (centered at mean). Models tested the interactions between maternal variables and infant age/sex. When an interaction was apparent, we conducted a joint F‐test to evaluate the joint significance of the main‐effect and interaction terms.

Dichotomous parameterizations of biomarker variables (e.g., iron deficiency anemia) represent conditions with meaningful biochemical or physiological consequences for human health. Continuous predictor infant age was centered to facilitate interpretation of the coefficients of other predictors, i.e. as their effects on FOLR1 at the mean infant age.

Control variables, which might be associated with both milk FOLR1 and maternal nutrition (Corbitt et al., 2019; Fujita, 2008; Fujita et al., 2019) included: maternal age and parity, postpartum resumption of menstruation (yes/no), breastfeeding frequency, complementary feeding status (yes/no), milk total protein concentration (measured by microBCA assay [Corbitt et al., 2019]), socioeconomic status (a dichotomous variable for low status by a combination of self‐reported poverty *and* below‐median household‐owned land size/livestock holding), and community. Since FOLR1 is a specific type of protein out of numerous proteins in milk (and highly correlated with total milk protein), we adjusted models for milk total protein to interpret the coefficients of the predictors as their effects on FOLR1 specifically, rather than their general associations with milk proteins. These variables were entered into regression models one at a time and retained if they either substantially altered one or more predictors' individual coefficients or improved model fit (evaluated with the Bayesian information criterion, BIC). If the entered variable did neither, then it was not retained in the model.

Resulting models were assessed for multicolinearity using variance inflation factor and other violations of regression assumptions using residual plots and Cook's distance for outlier influence/leverage. We report raw and standardized regression coefficients (β) from the models, and use a probability less than 0.10 as the evidence for association, favoring the power of type II error (Kim & Choi, 2021) because interaction terms can lose power when categorical predictors have relatively few 1 s (vs. 0 s) (UCLA Statistical Consulting Group, 2021). For ease of interpretation, we describe the effect size as the percent change in milk FOLR1 in the original linear scale, calculated by taking the exponent of the predicted values in log scale. Statistical analyses were carried out using Stata version 15.

## 3 RESULTS

### 3.1 Sample characteristics

The mean ± SD milk FOLR1 content was 610 ± 190 ng/ml (20.5 ± 6.4 nmol/L), ranging from 254 to 1248 ng/ml (8.5 to 41.9 nmol/L) (Table 1). The mean ± SD maternal age was 28 ± 7 years, ranging from 18 to 46; parity was 3.7 ± 2.3, ranging from 1 to 12; and BMI was 19.75 ± 2.9, ranging from 14.4 to 33.5. Of 203 mothers, 33% (*n* = 66) were underweight; 18% (*n* = 36) had iron deficiency anemia; 14% (*n* = 28) and 8% (*n* = 16) had hyperhomocysteinemia with homocysteine >12 and >14 μmol/L cutoff points respectively; and 17% (*n* = 35) had elevated inflammation. Three mothers had concurrent iron deficiency anemia and hyperhomocysteinemia (either cutoff); five had concurrent iron deficiency anemia and inflammation; and four and five had concurrent inflammation and hyperhomocysteinemia (per lower and higher cutoffs), respectively. The Pearson *χ* ^2^ tests and the Fisher's exact tests revealed that these co‐occurrences were no more likely than by chance alone. No mothers had three‐way co‐morbidity. The mean infant age was 8.1 ± 4.5 months, ranging from 0.8 to 19.5. The percentage of female and male infants was 45% (*n* = 91) and 55% (*n* = 112), respectively.

**TABLE 1:** Sample characteristics (*n* = 203)

	Mean or *n*	SD or %	Range
Milk			
Folate receptor‐α (FOLR1, ng/ml)	609.6	190.4	254, 1248
FOLR1 (ln‐transformed)	6.37	0.31	5.54, 7.13
Total protein (g/l)	0.99	0.22	0.65, 3.00
Total protein (ln‐transformed)	−0.03	0.19	−0.43, 1.10
Mother			
Age (year)	28.04	6.87	18, 46
Parity	3.65	2.25	1, 12
Body mass index	19.75	2.86	14.4, 33.5
Hemoglobin (Hb, g/dl)	12.94	1.78	7.3, 16.8
DBS soluble transferrin receptor (sTFR, mg/l)	4.94	2.44	2.3, 19.5
Serum homocysteine (μmol/l)	8.92	3.23	3.1, 21.3
Serum C‐reactive protein (mg/l)	3.91	6.88	0.03, 28.3^a^
Underweight (body mass index <18.5 kg/m^2^)	66	33	
Iron deficiency anemia (Hb <12 g/dl and sTfR >5 mg/L)	36	18	
Hyperhomocysteinemia (Homocysteine >12 μmol/L)	28	14	
Hyperhomocysteinemia (Homocysteine >14 μmol/L)	16	8	
Inflammation/infection (C‐reactive protein >5 mg/L)	35	17	
Infant			
Age (months)	8.13	4.50	0.8, 19.5
Centered infant age (months)	0.00	4.50	−7.3, 11.4
Female	91	45	
Male	112	55	
Feeding			
Breastfeeding frequency (reported bouts per 24 h)	9.23	4.13	3, 30
Exclusive breastfeeding	70	35	
Partial breastfeeding	132	65	

^a^ Assay's upper limit of detection.

### 3.2 Regression models

#### 3.2.1 Milk FOLR1 by maternal nutritional/inflammation status

Regression models are summarized in Table 2. Adjusted for milk total protein, milk FOLR1 did not differ by maternal underweight, iron deficiency anemia, or inflammation/infection. In the main‐effect models (1a, 1b), hyperhomocysteinemia was positively associated with milk FOLR1 (*p* < 0.016, *p* 0.035). Using the >12 and >14 μmol/L homocysteine cut‐point for hyperhomocysteinemia, predicted milk FOLR1 content was on average 14 and 15% higher among mothers with hyperhomocysteinemia than those with normal homocysteine.

**TABLE 2:** Regression models for milk folate receptor‐α (FOLR1, log‐transformed) by maternal nutritional/inflammation status and infant characteristics using lower (A) and higher (B) hyperhomocysteinemia cutoffs

A. Outcome milk FOLR1 (ln) *n* = 203												
	Model 1a				Model 2a				Model 3a			
Predictors	Coef.	*β*	SE	*p*	Coef.	*β*	SE	*p*	Coef.	*β*	SE	*p*
Underweight	0.063	0.097	0.036	0.081	0.052	0.079	0.035	0.141	0.066	0.102	0.036	0.067
Iron deficiency anemia	−0.016	−0.020	0.045	0.716	−0.028	−0.035	0.044	0.521	−0.019	−0.024	0.045	0.666
HHcy	0.119	0.134	0.049	0.016	0.109	0.123	0.048	0.023	0.052	0.058	0.069	0.457
Inflammation	0.029	0.035	0.044	0.518	0.018	0.022	0.043	0.681	0.022	0.028	0.045	0.614
Age	−0.024	−0.351	0.004	0.000	−0.032	−0.468	0.004	0.000	−0.024	−0.348	0.004	0.000
Sex (male)	−0.051	−0.082	0.034	0.135	−0.058	−0.095	0.033	0.077	−0.069	−0.112	0.036	0.059
Underweight × age					0.031	0.229	0.008	0.000				
HHcy × sex									0.133	0.110	0.098	0.177
Milk total protein (ln)	0.710	0.436	0.094	0.000	0.717	0.441	0.091	0.000	0.724	0.445	0.095	0.000
Constant	6.374		0.031	0.000	6.381		0.030	0.000	6.385		0.032	0.000
Model *P*	0.000				0.000				0.000			
*R* ^2^	0.422				0.460				0.428			
Adjusted *R* ^2^	0.402				0.438				0.404			
Mean VIF	1.06				1.14				1.36			

B. Outcome milk FOLR1 (ln) *n* = 203												
	Model 1b				Model 2b				Model 3b			
Predictors	Coef.	*β*	SE	*p*	Coef.	*β*	SE	*p*	Coef.	*β*	SE	*p*
Underweight	0.061	0.094	0.036	0.091	0.050	0.077	0.035	0.155	0.060	0.092	0.036	0.095
Iron deficiency anemia	−0.025	−0.032	0.045	0.573	−0.036	−0.045	0.044	0.408	−0.029	−0.037	0.045	0.513
HHcy	0.133	0.117	0.063	0.035	0.117	0.103	0.061	0.055	0.023	0.020	0.088	0.799
Inflammation	0.024	0.029	0.045	0.598	0.013	0.016	0.043	0.761	0.017	0.020	0.045	0.710
Age	−0.024	−0.357	0.004	0.000	−0.032	−0.474	0.004	0.000	−0.025	−0.360	0.004	0.000
Sex (male)	−0.052	−0.084	0.034	0.127	−0.059	−0.097	0.033	0.072	−0.069	−0.113	0.035	0.049
Underweight × age					0.031	0.229	0.008	0.000				
HHcy × sex									0.220	0.140	0.124	0.078
Milk total protein (ln)	0.707	0.435	0.094	0.000	0.714	0.439	0.092	0.000	0.712	0.437	0.094	0.000
Constant	6.384		0.03	0.000	6.391		0.029	0.000	6.396		0.031	0.000
Model *P*	0.000				0.000				0.000			
*R* ^2^	0.419				0.456				0.428			
Adjusted *R* ^2^	0.398				0.434				0.404			
Mean VIF	1.05				1.14				1.33			

Abbreviations: HHcy, hyperhomocysteinemia (serum homocysteine >12 and >14 μmoL/L for A and B, respectively); VIF, variance inflation factor.

#### 3.2.2 Milk FOLR1 by infant characteristics

In the main effect models, infant age was inversely associated with milk FOLR1 (*p* < 0.001 in both models 1a, 1b), predicting a 2% decrease per month. Male infant sex was unassociated with milk FOLR1 in these models.

#### 3.2.3 Milk FOLR1 by mother‐infant interaction

**FIGURE 2:**
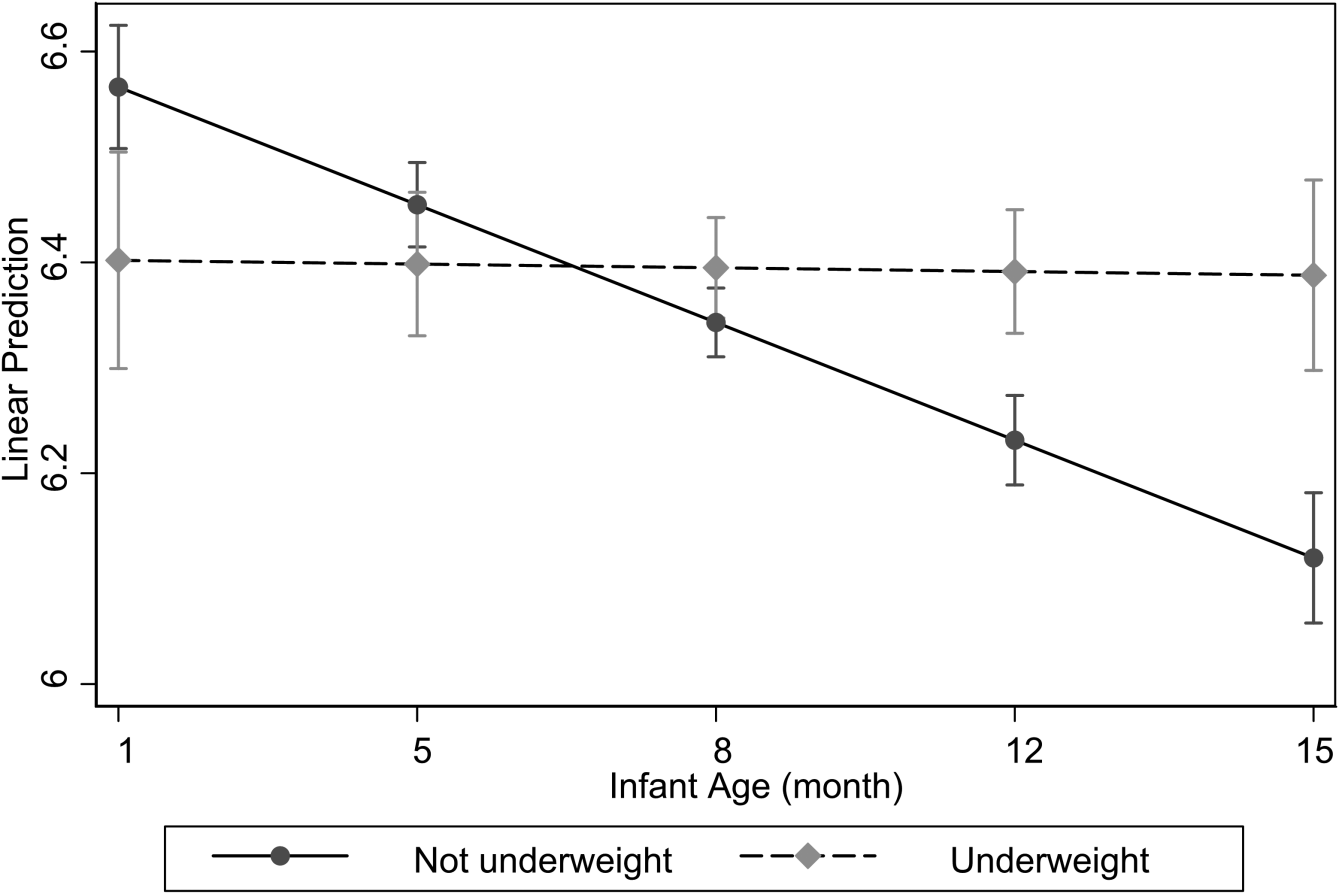
Margins plot displaying the effect of maternal underweight on predicted milk folate receptor‐α (FOLR1, ln‐transformed) across infant age per Model 2a. Bars indicate 90% CIs

Of the eight possible interactions, two were noteworthy (Table 2 interaction models 2 and 3; all other interaction models are shown in Supplemental Information, Tables S1 and S2). First, there was an interaction (*p* < 0.001) between infant age and maternal underweight (Joint *F* test *F*_(2,194)_ 8.47, *p* 0.0003, Model 2a; Joint *F* test *F*_(2,194)_ 8.26, *p* 0.0004, Model 2b). Figure 2 illustrates the nature of this interaction. Among underweight mothers, FOLR1 was unassociated with infant age, while among mothers not underweight, milk FOLR1 declined with infant age. Underweight mothers maintained delivery of milk FOLR1 albeit with a slight decrease as infant age advanced (<1% per month); mothers not underweight delivered steeply diminishing milk FOLR1 with advancing infant age (11% per month).

**FIGURE 3:**
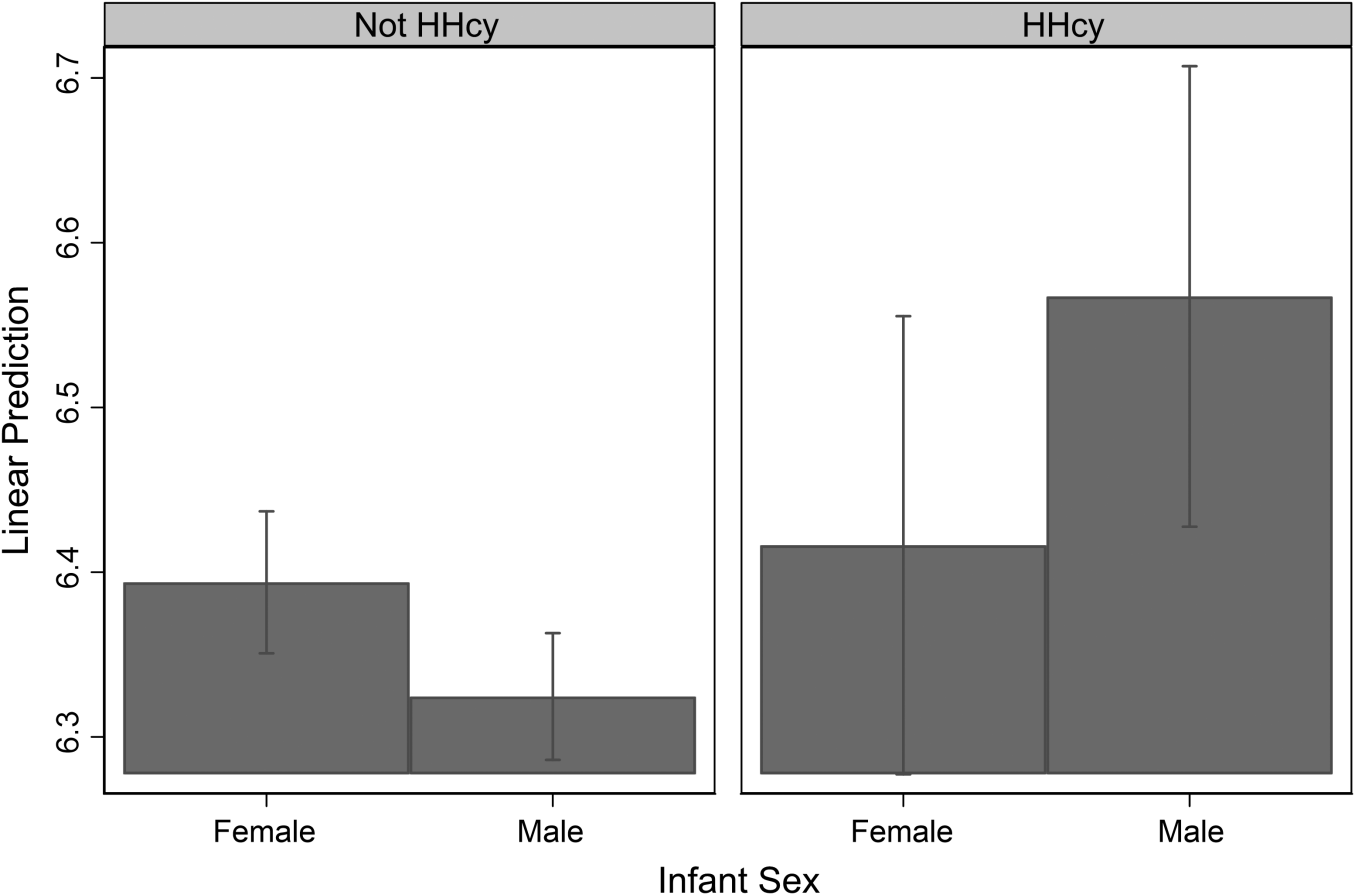
Predictive margins of hyperhomocysteinemia (HHcy) on milk folate receptor‐α (FOLR1, lntransformed) by infant sex per Model 3b. Bars indicate 90% CIs

Second, there was an interaction (*p* 0.078) between infant sex and hyperhomocysteinemia per the >14 μmol/L cutoff (Joint *F* test *F*(_2,194_) 2.76, *p* 0.066, Model 3b). The inclusion of this interaction altered the coefficients of main effects as well; an inverse association between male infant sex and FOLR1 became apparent (*p* 0.049) while the association between hyperhomocysteinemia and FOLR1 diminished. The pattern of this interaction is illustrated in Figure 3. Milk folate delivery was higher for sons than daughters (the difference of 11%) among mothers with hyperhomocysteinemia, while among mothers without hyperhomocysteinemia, milk FOLR1 delivery was higher for daughters than sons (the difference of 7%).

## 4 DISCUSSION

We used data from northern Kenya to investigate whether maternal delivery of folate to milk may be buffered against maternal nutritional and infectious disease stress and proactively calibrated to protect infants against similar stresses. We tested a set of hypotheses combining the ideas of maternal buffering and maternal protective effort. We expected milk folate to be generally buffered against maternal nutritional or disease stress, but we further expected that the extent of buffering would vary in ways reflecting the balance between cost and benefit of buffering (Figure 1). Namely, we generally predicted that mothers raising younger or male infants would deliver more folate to milk, particularly among mothers with nutritional deficiencies or inflammation/infection, who face the highest costs to buffering milk folate. Our results did not fully conform to the pattern of variation we expected; however, we did observe patterns that are indicative of maternal buffering and protective efforts, particularly for infants with elevated needs (i.e. younger/male). The interactive effects we observed may suggest a more complex relationship between maternal condition and infant vulnerability than we predicted.

### 4.1 Buffered milk folate (mothers maintain, or increase, folate delivery to milk)

Our findings generally support the maternal buffering hypothesis ‐ using FOLR1 as a measure of folate, milk folate was unaffected by maternal underweight, iron deficiency anemia, or inflammation, and was *positively* associated with hyperhomocysteinemia (likely representing folate deficiency). The lack of evidence for compromised milk folate delivery among mothers with underweight, iron deficiency anemia, infection, or hyperhomocysteinemia suggests that mothers of northern Kenya during a serious drought and resulting food scarcity generally buffered milk folate against their own nutritional and disease stress—mothers could maintain their folate delivery to milk regardless of malnutrition or inflammation/infection. This extends the evidence for maternal buffering of human milk beyond energy content (Fujita, Paredes Ruvalcaba, Wander, et al., 2018; Lönnerdal, 1986; Mandel et al., 2005). This also corroborates nutritional scientists' characterization of milk folate as a vitamin largely immune to decrease even when mothers are deficient (Allen, 2012).

Our results further contribute to the literature with a new discovery that milk folate is *positively* associated with hyperhomocysteinemia. This has two possible (and not mutually exclusive) interpretations: Mothers may have developed hyperhomocysteinemia as a consequence of delivering folate to milk. Alternatively, mothers may increase delivery of folate to milk when their dietary folate (and/or related B‐vitamins) are in shortfall, providing infants who are likely to experience folate deficiency in the future with an abundance of folate when the opportunity (lactation) is available. This may improve the child's survival in a nutrient‐limited environment after the period of breastfeeding. The present study, with a cross‐sectional research design, is unable to distinguish between these explanations.

### 4.2 Buffering and protection (mothers conditionally increase/decrease folate delivery to milk)

We also found two interactions between maternal and infant factors in predicting milk folate content. Among underweight mothers, milk folate changed little with infant age, while among non‐underweight mothers, milk folate declined with infant age. Among mothers with hyperhomocysteinemia, milk folate content appeared to be higher for male infants. These interactions suggest that mothers' buffering of milk folate may decrease or increase when nutritional stress is combined with certain infant characteristics indicative of their vulnerability.

We had predicted a decline in milk folate with infant age among mothers experiencing nutritional or infectious disease stress (representing more buffering for infants with greater needs and attenuated buffering for older infants with less needs when buffering was most costly). Instead, we found that, among underweight mothers, milk folate content was constant with infant age, while among non‐underweight mothers, milk folate content declined with infant age, albeit starting with high content. This is consistent with the “protective” component of our predictions—that milk folate would be higher for younger infants, who are more vulnerable to failures due to folate deficiency. However, it is inconsistent with our expectation that among mothers paying the highest costs to deliver folate to milk (those with limited intake, or elevated usage, of folate due to nutritional or infectious disease stress), buffering would be attenuated for less vulnerable infants. Declining milk folate with age was a consistent pattern across models. It is striking that this is not apparent among underweight mothers.

One explanation for prolonged high (i.e. constant) milk folate among underweight mothers may be that it compensates for lower folate delivery during the initial postpartum period, when undernourished mothers may have had limited nutrient reserves (having spent much of them, including folate, to carry pregnancy to term). Alternatively, it may represent a delayed shift to maternal investment in future (vs. current) reproduction (Hill & Kaplan, 1999; Stearns, 1992) among underweight mothers. We should note that predicted milk FOLR1 values for underweight mothers in the early postpartum period are likely to be based on few cases (as pregnancy weight gain makes it unlikely that even an undernourished mother would fall under BMI of 18.5, the definition for underweight, in the early postpartum weeks), and so differences in FOLR1 among mothers at young infant ages should be interpreted with caution. However, distinct patterns became apparent at later infant ages: among non‐underweight (better nourished) mothers milk folate decreased with infant age.

We also found some evidence that milk folate might have been considerably higher for mothers with hyperhomocysteinemia who were raising sons, compared to daughters. This may represent protection for male infants, who may be, relative to female infants, more susceptible to infectious diseases (Naeye et al., 1971) and sensitive to environmental stress during growth (Stinson, 1985). In the context of drought and heightened nutritional and disease stress in northern Kenya, these protective benefits may outweigh the cost to the mother (e.g., folate deficiency).

### 4.3 When mothers may increase milk folate delivery/buffering: cost–benefit analysis

These interactions in the mother‐infant dyads suggest that maternal condition may be sacrificed for milk folate delivery during prolonged nutritional stress (with likely additional stressors from this environment, such as infectious diseases). We found that maternal delivery of milk folate did not decrease with infant age among underweight mothers as it did among those who were normal weight, and that maternal delivery of milk folate was higher among folate deficient mothers (especially for sons), than among those who were folate replete. This may represent elevated maternal effort to protect particularly vulnerable infants against folate deficiency – these mothers may have prioritized infant health and survival over their own needs. Such altruism would be unreasonable if it risked maternal mortality, which could jeopardize infant survival, for human infants are highly altricial and require maternal care; however, it would be reasonable so long as maternal or infant survival and well‐being are not hindered.

In environments with prolonged, or repeated, severe droughts and famines such as the one our study participants faced, food insecurity can drastically diminish the prospect of successful future reproduction, given the large amount of maternal resource that must be transferred, including gestational and lactational transfer of nutrients such as folate. In this context of resource scarcity, mothers may thus decrease allocation of folate to future reproduction and instead increase allocation to the current infant. The benefit of additional investment in the infant (i.e. survival) likely outweighs the opportunity cost for the mother (forfeited future offspring). This is particularly likely when infants have heightened vulnerability to early death. Infants of underweight mothers, who may have experienced suboptimal intrauterine environment and nutrient/hormone transfer that may have long‐term effects (Henriksen & Clausen, 2002; Hoffman et al., 2019) likely benefit from elevated milk folate delivery for physiological processes involved in their health, growth, and development.

### 4.4 Study limitations

Our study has a few limitations. Some limitations are due to the use of a cross‐sectional data set. First, cross‐sectional data do not allow us to address the causal direction. For instance, we found that maternal hyperhomocysteinemia was associated with higher milk folate. While we are inclined to interpret this as a reflection of elevated folate delivery causing mothers to develop folate deficiency and therefore hyperhomocysteinemia, it is also possible that mothers preemptively increased folate delivery to milk to protect infants against the nutritional stress underlying their hyperhomocysteinemia.

Second, the predicted change in FOLR1 across the cross‐sectional infant age may be an imperfect surrogate for the longitudinal change that may occur if some unaccounted factors affected FOLR1 in an age‐dependent fashion. For instance, diurnal patterns in FOLR1 might have dissipated (or expanded) across postpartum age, as reported among well‐nourished US mothers (Udipi et al., 1987). If so, the cross‐sectional association between FOLR1 and infant age would represent the combined longitudinal changes due to advancing infant age and dissipating diurnality. Longitudinal research designs are needed to clarify causal directions (particularly with regard to higher milk folate among mothers with hyperhomocysteinemia).

Our analyses were also limited by the number of hyperhomocysteinemia cases by the >14 μmol/L homocysteine cut‐point (*n* = 16). We may have missed real but subtle patterns in these models.

Another possible limitation relates to the long‐term storage of milk specimens, which may have affected FOLR1 concentrations. This is, however, unlikely—a study indicated that the analytic recovery of folate in blood serum stored for 29 years at −25°C was approximately 80% (Hannisdal et al., 2010). FOLR1 is more robust than folate, and the present project utilized specimens stored for a shorter duration (11 years) at substantially lower temperatures <−70°C, more favorable for minimizing the long‐term storage effect on biological specimens. FOLR1 also tolerates multiple (at least three) freeze–thaw cycles when frozen cryogenically (Balion, 2011).

In interpreting the results, the practical importance of the effect size should ideally be appraised against what may be a biologically meaningful effect size (Wasserstein et al., 2019). The paucity of information on human milk FOLR1 (because few previous studies quantified FOLR1 specifically) presents a challenge in this regard. However, the broader literature on blood FOLR1 concentrations provides a general benchmark to gauge the possible biological meaning of differences in milk FOLR1 we report in this study. A 22% difference in blood serum FOLR1 concentration has been attributed to active periodontitis vs. healthy control (Alkan et al., 2019), while a difference of 34% has been observed between mothers who did (vs. did not) experience a neural tube defect birth (Celik et al., 2014). Gauged against these values, the magnitude of effects we reported here (e.g., 7%–15%) may have important implications for infants' health, growth and development long term.

Finally, we have used FOLR1 as a biomarker for milk folate, rather than measuring milk folate itself. This is justified because milk folate is less robust to storage; however, it does introduce opportunity for error if the association between milk folate and FOLR1 does not hold under some conditions.

### 4.5 Future directions

Studies on buffering of milk nutrient content, including folate, are needed to understand whether and under which contexts buffering occurs and to what extent. Such studies need to investigate communities subject to varying degrees of nutritional stress, including severe stresses such as those of a prolonged drought. Furthermore, future study is needed to understand whether the magnitude of the effects we report here are biologically meaningful, and how maternal buffering relates to folate status in nursing infants. Investigation of interactions in the mother‐infant dyad are a necessary step toward better understanding of the impact of context on nutrition interventions (Raiten et al., 2021).

## 5 CONCLUSIONS

Our findings support the maternal buffering hypothesis, and reveal complex interactions between maternal and infant factors in milk folate content. Mothers generally seem to buffer milk folate content against their own nutritional or disease stress. Milk folate does vary with infant characteristics (age and sex) in ways arguably congruent with the protective hypothesis. Further research is needed to clarify the diachronic dimension in milk folate variation, such as whether elevated maternal delivery of milk folate may be triggered by, or contribute to, maternal folate deficiency, and to determine the impact of milk folate on infant folate nutrition and health, growth, and development outcomes.

## AUTHOR CONTRIBUTIONS

**Masako Fujita:** Conceptualization (lead); data curation (lead); formal analysis (lead); funding acquisition (lead); investigation (lead); methodology (lead); project administration (lead); resources (lead); software (lead); supervision (lead); validation (lead); visualization (lead); writing – original draft (equal); writing – review and editing (equal). **Katherine Wander:** Conceptualization (equal); formal analysis (equal); writing – original draft (equal); writing – review and editing (equal). **Tin Tran:** Formal analysis (supporting); investigation (supporting); resources (supporting); writing – original draft (supporting); writing – review and editing (supporting). **Eleanor Brindle:** Data curation (supporting); writing – review and editing (supporting).

## FUNDING INFORMATION

This work was supported by National Science Foundation (M.F., grant numbers BCS 0622358, BCS 1638167) and the Wenner‐Gren Foundation (M.F., grant numbers 7460, 9278). Eunice Kennedy Shriver National Institute of Child Health and Human Development research infrastructure grant (P2C HD042828) supported E.B., and the Summer Research Opportunity Program of Michigan State University supported T.T. during a portion of the research.

## CONFLICT OF INTEREST

The authors declare no conflict of interest.

## Supporting information


**TABLE S1** Regression models for milk folate receptor‐α (FOLR1, log‐transformed) with an interaction term for infant age having >0.1 probability, using lower (*A*) and higher (*B*) hyperhomocysteinemia cutoffs.

**TABLE S2** Regression models for milk folate receptor‐α (FOLR1, log‐transformed) with an interaction term for infant sex having >0.1 probability, using lower (*A*) and higher (*B*) hyperhomocysteinemia cutoffs.

## Data Availability

The data utilized for this study are available at the Zenodo open access repository under doi: 10.5281/zenodo.5527188 (Fujita, 2021).

## References

[ajpa24603-bib-0001] Alkan, D. , Guven, B. , Turer, C. C. , Balli, U. , & Can, M. (2019). Folate‐receptor 1 level in periodontal disease: A pilot study. BMC Oral Health, 19(1), 5. 10.1186/s12903-019-0909-z 31604439 PMC6787999

[ajpa24603-bib-0002] Allen, L. H. (2012). B vitamins in breast milk: Relative importance of maternal status and intake, and effects on infant status and function. Advances in Nutrition, 3(3), 362–369. 10.3945/an.111.001172 22585913 PMC3649471

[ajpa24603-bib-0003] Aparicio‐Ugarriza, R. , Palacios, G. , Alder, M. , & Gonzalez‐Gross, M. (2015). A review of the cut‐off points for the diagnosis of vitamin B‐12 deficiency in the general population. Clinical Chemistry and Laboratory Medicine, 53(8), 1149–1159. 10.1515/cclm-2014-0784 25470607

[ajpa24603-bib-0004] Bailey, L. B. , & Gregory, J. F. (1999). Folate metabolism and requirements. Journal of Nutrition, 129(4), 779–782. 10203550 10.1093/jn/129.4.779

[ajpa24603-bib-0005] Baily, L. B. (1998). Dietary reference intakes for folate: The debut of dietary folate equivalents. Nutrition Reviews, 56(10), 294–299. 9810807 10.1111/j.1753-4887.1998.tb01662.x

[ajpa24603-bib-0006] Balion, C. (2011). Clinical utility of serum and red blood cell analysis. Folate: Clinical Laboratory News, 37(1), 8–10.

[ajpa24603-bib-0007] Bartley, K. A. , Underwood, B. A. , & Deckelbaum, R. J. (2005). A life cycle micronutrient perspective for women's health. American Journal of Clinical Nutrition, 81(5), 1188S–1193S. 15883450 10.1093/ajcn/81.5.1188

[ajpa24603-bib-0008] Black, M. M. (2008). Effects of B‐12 and folate deficiency on brain development in children. Food and Nutrition Bulletin, 29(2), S126–S131. 10.1177/15648265080292s117 18709887 PMC3137939

[ajpa24603-bib-0009] Breakey, A. A. , Hinde, K. , Valeggia, C. R. , Sinofsky, A. , & Ellison, P. T. (2015). Illness in breastfeeding infants relates to concentration of lactoferrin and secretory immunoglobulin a in mother's milk. Evolution Medicine and Public Health, 1, 21–31. 10.1093/emph/eov002 PMC4334701 25608691

[ajpa24603-bib-0010] Brindle, E. , Fujita, M. , Shofer, J. , & O'Connor, K. A. (2010). Serum, plasma, and dried blood spot high‐sensitivity C‐reactive protein enzyme immunoassay for population research. Journal of Immunological Methods, 362(1–2), 112–120. 10.1016/j.jim.2010.09.014 20850446 PMC2964394

[ajpa24603-bib-0011] Celik, E. , Karaer, A. , Turkcuoglu, I. , Turhan, U. , Gungoren, A. , Taskapan, C. , Ozyalin, F. , & Berker, B. (2014). Association of folic acid receptor alpha in maternal serum with neural tube defects. The Journal of Maternal‐Fetal & Neonatal Medicine, 27(11), 1083–1087. 10.3109/14767058.2013.849239 24094304

[ajpa24603-bib-0012] Cooperman, J. M. , Dweck, H. S. , Newman, L. J. , Garbarino, C. , & Lopez, R. (1982). THE folate in human‐milk. American Journal of Clinical Nutrition, 36(4), 576–580. 6896957 10.1093/ajcn/36.4.576

[ajpa24603-bib-0013] Corbitt, M. , Paredes Ruvalcaba, N. , & Fujita, M. (2019). Variation in breast milk macronutrient contents by maternal anemia and hemoglobin concentration in northern Kenya. American Journal of Human Biology, 31(3), e23238. 10.1002/ajhb.23238 30908793

[ajpa24603-bib-0014] Courtemanche, C. , Elson‐Schwab, I. , Mashiyama, S. T. , Kerry, N. , & Ames, B. N. (2004). Folate deficiency inhibits the proliferation of primary human CD8(+) T lymphocytes in vitro. Journal of Immunology, 173(5), 3186–3192. 10.4049/jimmunol.173.5.3186 15322179

[ajpa24603-bib-0015] Dancheck, B. , Nussenblatt, V. , Ricks, M. O. , Kumwenda, N. , Neville, M. C. , Moncrief, D. T. , Taha, T. E. , & Semba, R. D. (2005). Breast milk retinol concentrations are not associated with systemic inflammation among breast‐feeding women in Malawi. The Journal of Nutrition, 135(2), 223–226. 15671217 10.1093/jn/135.2.223

[ajpa24603-bib-0016] Donangelo, C. M. , Trugo, N. M. F. , Koury, J. C. , Barretosilva, M. I. , Freitas, L. A. , Feldheim, W. , & Barth, C. (1989). Iron, zinc, folate and vitamin‐B12 nutritional‐status and milk‐composition of low‐income Brazilian mothers. European Journal of Clinical Nutrition, 43(4), 253–266. 2661218

[ajpa24603-bib-0017] Ford, J. E. (1974). Some observations on possible nutritional significance of vitamin‐B12‐binding and folate‐binding proteins in milk. British Journal of Nutrition, 31(2), 243–257. 10.1079/bjn19740030 4595172

[ajpa24603-bib-0018] Fratkin, E. (1998). Ariaal pastoralists of Kenya: Surviving drought and development in Africa's arid lands(p. 139).

[ajpa24603-bib-0019] Fratkin, E. , & Roth, E. A. (1990). Drought and economic differentiation among Ariaal pastoralists of Kenya. Human Ecology, Needham Heights, MA: Allyn & Bacon Inc. 18(4), 385–402. 10.1007/bf00889464 12285380

[ajpa24603-bib-0020] Fratkin, E. , Roth, E. A. , & Nathan, M. A. (2004). Pastoral sedentarization and its effects on children's diet, health, and growth among Rendille of northern Kenya. Human Ecology, 32(5), 531–559. 10.1007/s10745-004-6096-8

[ajpa24603-bib-0021] Freedman, B. (2001). Cytomegalovirus seropositivity and C‐reactive protein have independent and combined predictive value for mortality in patients with angiographically demonstrated coronary artery disease. Circulation, 104(5), E20–E21. 10.1161/01.CIR.104.5.e20 11479266

[ajpa24603-bib-0022] Fujita, M. (2003). Sedentarization, seasonality, and economic differentiation: Maternal diet and health in Ariaal‐Rendille communities in northern Kenya. In Anthropology. University of Victoria.

[ajpa24603-bib-0023] Fujita, M. (2008). An epidemiological and evolutionary investigation of mother‐offspring vitamin a transfer [Unpublished PhD thesis]. University of Washington, Seattle, WA.

[ajpa24603-bib-0024] Fujita, M. (2021). Ariaal Milk FOLR1 Data (Version 1) [Data set]. Zenodo. 10.5281/zenodo.5527188.

[ajpa24603-bib-0025] Fujita, M. , Brindle, E. , Lo, Y.‐J. , Castro, P. , & Cameroamortegui, F. (2014). Nutrient intakes associated with elevated serum C‐reactive protein concentrations in normal to underweight breastfeeding women in northern Kenya. American Journal of Human Biology, 26(6), 796–802. 10.1002/ajhb.22600 25130535 PMC5298890

[ajpa24603-bib-0026] Fujita, M. , Lo, Y.‐J. , & Baranski, J. R. (2012). Dietary diversity score is a useful indicator of vitamin a status of adult women in northern Kenya. American Journal of Human Biology, 24(6), 829–834. 10.1002/ajhb.22327 23015415

[ajpa24603-bib-0027] Fujita, M. , Lo, Y.‐J. , & Brindle, E. (2017). Nutritional, inflammatory, and ecological correlates of maternal retinol allocation to breast milk in agro‐pastoral Ariaal communities of northern Kenya. American Journal of Human Biology, 29(4):e22961. 10.1002/ajhb.22961 PMC5511767 28094879

[ajpa24603-bib-0028] Fujita, M. , Paredes Ruvalcaba, N. , & Corbitt, M. (2018). The evolutionary ecology of breastmilk folate among Ariaal agro‐pastoralists in Kenya. American Journal of Physical Anthropology, 165, 91.

[ajpa24603-bib-0029] Fujita, M. , Paredes Ruvalcaba, N. , Wander, K. , Corbitt, M. , & Brindle, E. (2018). Buffered or impaired: Maternal anemia, inflammation and breast milk macronutrients in northern Kenya. American Journal of Physical Anthropology, 168(2), 329–339. 10.1002/ajpa.23752 30575959 PMC6352968

[ajpa24603-bib-0030] Fujita, M. , Roth, E. A. , Nathan, M. A. , & Fratkin, E. (2004). Sedentism, seasonality, and economic status: A multivariate analysis of maternal dietary and health statuses between pastoral and agricultural Ariaal and Rendille communities in northern Kenya. American Journal of Physical Anthropology, 123(3), 277–291. 10.1002/ajpa.10310 14968423

[ajpa24603-bib-0031] Fujita, M. , Roth, E. A. , Nathan, M. A. , & Fratkin, E. (2005). Sedentarization and seasonality: Maternal dietary and health consequences in Ariaal and Rendille communities in northern Kenya. In Fratkin E and E Roth (Eds.) As pastoralists settle: Social, health, and economic consequences of pastoral sedentarization in Marsabit District, Kenya. (Vol. 1, pp. 209–234). New York: Kluwer Academic/Plenum Publishers.

[ajpa24603-bib-0032] Fujita, M. , Shell‐Duncan, B. , Ndemwa, P. , Brindle, E. , Lo, Y.‐J. , Kombe, Y. , & O'Connor, K. A. (2011). Vitamin a dynamics in breastmilk and liver stores: A life history perspective. American Journal of Human Biology, 23(5), 664–673. 10.1002/ajhb.21195 21695742 PMC3938187

[ajpa24603-bib-0033] Fujita, M. , & Wander, K. (2017). A test of the optimal iron hypothesis among breastfeeding Ariaal mothers in northern Kenya. American Journal of Physical Anthropology, 164(3), 586–597. 10.1002/ajpa.23299 28832929

[ajpa24603-bib-0034] Fujita, M. , Wander, K. , Paredes Ruvalcaba, N. , & Brindle, E. (2019). Human milk sIgA antibody in relation to maternal nutrition and infant vulnerability in northern Kenya. Evolution Medicine and Public Health, 1, 201–211. 10.1093/emph/eoz030 PMC7216193 32405414

[ajpa24603-bib-0090] González‐Fernández, D., Pons, E. del C., Rueda, D., Sinisterra, O. T., Murillo, E., Scott, M. E., & Koski, K. G. (2017). C‐reactive protein is differentially modulated by co‐existing infections, vitamin deficiencies and maternal factors in pregnant and lactating indigenous Panamanian women. Infectious Diseases of Poverty, 6(1). 10.1186/s40249-017-0307-1 PMC5455098 28571565

[ajpa24603-bib-0035] Gorelova, V. , Bastien, O. , De Clerck, O. , Lespinats, S. , Rebeille, F. , & Van Der Straeten, D. (2019). Evolution of folate biosynthesis and metabolism across algae and land plant lineages. Scientific Reports, 9, 5731. 10.1038/s41598-019-42146-5 30952916 PMC6451014

[ajpa24603-bib-0036] Green, R. (2011). Indicators for assessing folate and vitamin B‐12 status and for monitoring the efficacy of intervention strategies. American Journal of Clinical Nutrition, 94(2), 666S–672S. 10.3945/ajcn.110.009613 21733877 PMC3142735

[ajpa24603-bib-0037] Hannisdal, R. , Gislefoss, R. E. , Grimsrud, T. K. , Hustad, S. , Morkrid, L. , & Ueland, P. M. (2010). Analytical recovery of folate and its degradation products in human serum stored at‐25 degrees C for up to 29 years. Journal of Nutrition, 140(3), 522–526. 10.3945/jn.109.116418 20071651

[ajpa24603-bib-0038] Henriksen, T. , & Clausen, T. (2002). The fetal origins hypothesis: Placental insufficiency and inheritance versus maternal malnutrition in well‐nourished populations. Acta Obstetricia et Gynecologica Scandinavica, 81(2), 112–114. 10.1034/j.1600-0412.2002.810204.x 11942899

[ajpa24603-bib-0039] Hill, K. , & Kaplan, H. (1999). Life history traits in humans: Theory and empirical studies. Annual Review of Anthropology, 28, 397–430. 10.1146/annurev.anthro.28.1.397 12295622

[ajpa24603-bib-0040] Hinde, K. , Power, M. L. , & Oftedal, L. T. (2009). Rhesus macaque milk: Magnitude, sources, and consequences of individual variation over lactation. American Journal of Physical Anthropology, 138(2), 148–157. 10.1002/ajpa.20911 18711734 PMC2615798

[ajpa24603-bib-0041] Hoffman, D. , Arts, M. , & Begin, F. (2019). The “first 1,000 days+” as key contributor to the double burden of malnutrition. Annals of Nutrition and Metabolism, 75(2), 99–102. 10.1159/000503665 31743897

[ajpa24603-bib-0042] Holm, J. , & Hansen, S. I. (2020). Characterization of soluble folate receptors (folate binding proteins) in humans. Biological roles and clinical potentials in infection and malignancy. Biochimica Et Biophysica Acta‐Proteins and Proteomics, 1868(10), 10. 10.1016/j.bbapap.2020.140466 32526472

[ajpa24603-bib-0043] Houghton, L. A. , Yang, J. , & O'Connor, D. L. (2009). Unmetabolized folic acid and total folate concentrations in breast milk are unaffected by low‐dose folate supplements. American Journal of Clinical Nutrition, 89(1), 216–220. 10.3945/ajcn.2008.26564 19056550

[ajpa24603-bib-0044] Jablonski, N. G. , & Chaplin, G. (2000). The evolution of human skin coloration. Journal of Human Evolution, 39(1), 57–106. 10.1006/jhev.2000.0403 10896812

[ajpa24603-bib-0045] Kenya National Bureau of Statistics , & ICF Macro . (2010). Kenya demographic and health survey 2008–09. KNBS and ICF Macro.

[ajpa24603-bib-0046] Khambalia, A. , Latulippe, M. E. , Campos, C. , MerloS, C. , Villalpando, S. , Picciano, M. F. , & O'Connor, D. L. (2006). Milk folate secretion is not impaired during iron deficiency in humans. Journal of Nutrition, 136(10), 2617–2624. 10.1093/jn/136.10.2617 16988136

[ajpa24603-bib-0047] Kim, J. H. , & Choi, I. (2021). Choosing the level of significance: A decision‐theoretic approach. Abacus‐A Journal of Accounting Finance and Business Studies, 57(1), 27–71. 10.1111/abac.12172

[ajpa24603-bib-0048] Lönnerdal, B. (1986). Effects of maternal dietary intake on human milk composition. Journal of Nutrition, 116(4), 499–513. 3514820 10.1093/jn/116.4.499

[ajpa24603-bib-0049] Lucock, M. , Beckett, E. , Martin, C. , Jones, P. , Furst, J. , Yates, Z. , Jablonski, N. G. , Chaplin, G. , & Veysey, M. (2017). UV‐associated decline in systemic folate: Implications for human nutrigenetics, health, and evolutionary processes. American Journal of Human Biology, 29(2):e22929. 10.1002/ajhb.22929 27771938

[ajpa24603-bib-0050] Mackey, A. D. , & Picciano, M. F. (1999). Maternal folate status during extended lactation and the effect of supplemental folic acid. American Journal of Clinical Nutrition, 69(2), 285–292. 9989694 10.1093/ajcn/69.2.285

[ajpa24603-bib-0051] Madan, E. , Haas, J. , Frongillo, E. , Menon, P. , Kumar, V. , Kumar, A. , Kumar, A. , Singh, S. , & Dixit, S. (2019). Monthly variation in velocities of weight and length growth in infants under six months of age in rural Uttar Pradesh, India. Current Developments in Nutrition, 3, 1. 10.1093/cdn/nzz034.P10-008-19

[ajpa24603-bib-0052] Mandel, D. , Lubetzky, R. , Dollberg, S. , Barak, S. , & Mimouni, F. B. (2005). Fat and energy contents of expressed human breast milk in prolonged lactation. Pediatrics, 116(3), E432–E435. 10.1542/peds.2005-0313 16140689

[ajpa24603-bib-0053] Marsabit District . (2006). Drought monitoring bulletin ‐ Marsabit District: Period April 2006.

[ajpa24603-bib-0054] Mason, J. B. , & Selhub, J. (1988). Folate‐binding protein and the absorption of folic‐acid in the small‐intestine of the suckling rat. American Journal of Clinical Nutrition, 48(3), 620–625. 2843025 10.1093/ajcn/48.3.620

[ajpa24603-bib-0055] McKay, J. A. , & Mathers, J. C. (2016). Maternal folate deficiency and metabolic dysfunction in offspring. The Proceedings of the Nutrition Society, 75(1), 90–95. 10.1017/S0029665115004280 26621202

[ajpa24603-bib-0056] Metz, J. (1970). Folate deficiency conditioned by lactation. American Journal of Clinical Nutrition, 23(6), 843–847. 5464396 10.1093/ajcn/23.6.843

[ajpa24603-bib-0057] Miller, E. M. (2011). Breastfeeding and immunity in Ariaal mothers and infants [Unpublished PhD thesis]. University of Michigan.

[ajpa24603-bib-0058] Miller, E. M. , Aiello, M. O. , Fujita, M. , Hinde, K. , Milligan, L. , & Quinn, E. A. (2013). Field and laboratory methods in human milk research. American Journal of Human Biology, 25(1), 1–11. 10.1002/ajhb.22334 23109280

[ajpa24603-bib-0059] Naeye, R. L. , Burt, L. S. , Wright, D. L. , Blanc, W. A. , & Tatter, D. (1971). Neonatal mortality, the male disadvantage. Pediatrics, 48(6), 902–906. 5129451

[ajpa24603-bib-0060] Nakagomi, A. , Ben Freedman, S. , & Geczy, C. L. (2000). Interferon‐gamma and lipopolysaccharide potentiate monocyte tissue factor induction by C‐reactive protein—Relationship with age, sex, and hormone replacement treatment. Circulation, 101(15), 1785–1791. 10.1161/01.cir.101.15.1785 10769278

[ajpa24603-bib-0061] Nygren‐Babol, L. , & Jagerstad, M. (2012). Folate‐binding protein in milk: A review of biochemistry, physiology, and analytical methods. Critical Reviews in Food Science and Nutrition, 52(5), 410–425. 10.1080/10408398.2010.500499 22369260

[ajpa24603-bib-0062] O'Connor, D. L. , Green, T. , & Picciano, M. F. (1997). Maternal folate status and lactation. Journal of Mammary Gland Biology and Neoplasia, 2(3), 279–289. 10882311 10.1023/a:1026388522182

[ajpa24603-bib-0063] Oconnor, D. L. , Picciano, M. F. , Sherman, A. R. , & Burgert, S. L. (1987). Depressed folate incorporation into milk secondary to iron‐deficiency in the rat. Journal of Nutrition, 117(10), 1715–1720. 3668685 10.1093/jn/117.10.1715

[ajpa24603-bib-0064] Paredes Ruvalcaba, N. , Bignall, E. , & Fujita, M. (2020). Age and socioeconomic status in relation to risk of maternal anemia among the Ariaal agropastoralists of northern Kenya. Human Ecology, 48(1), 47–54. 10.1007/s10745-020-00129-5

[ajpa24603-bib-0065] Prentice, A. , Prentice, A. M. , & Goldberg, G. R. (1994). Body mass index and lactation performance. European Journal of Clinical Nutrition, 48, S78–S86. 7843163

[ajpa24603-bib-0066] Prentice, A. , Prentice, A. M. , & Whitehead, R. G. (1981). Breast‐milk fat concentrations of rural African women 0.1. Short‐term variations within individuals. British Journal of Nutrition, 45(3), 483–494. 10.1079/bjn19810127 7195279

[ajpa24603-bib-0067] Prentice, A. , Watkinson, M. , Prentice, A. M. , Cole, T. J. , & Whitehead, R. G. (1984). Breast‐milk antimicrobial factors of rural Gambian mothers 0.2. Influence of season and prevalence of infection. Acta Paediatrica Scandinavica, 73(6), 803–809. 10.1111/j.1651-2227.1984.tb17779.x 6543089

[ajpa24603-bib-0068] R&D . (2011). Quantikine ELISA: Human FOLR1 immunoassay, catalog number DFLR10.

[ajpa24603-bib-0069] Raiten, D. J. , Combs, G. F. , Steiber, A. L. , & Bremer, A. A. (2021). Perspective: Nutritional status as a biological variable (NABV): Integrating nutrition science into basic and clinical research and care. Advances in Nutrition, 12, 1599–1609. 10.1093/advances/nmab046 34009250 PMC8483963

[ajpa24603-bib-0070] Rosenberg, I. H. , & Selhub, J. (2006). Folate and vitamin B12 transport systems in the developing infant. Journal of Pediatrics, 149(5), S62–S63. 10.1016/j.jpeds.2006.06.053

[ajpa24603-bib-0071] Salmenpera, L. , Perheentupa, J. , & Siimes, M. A. (1986). Folate nutrition is optimal in exclusively breast‐fed infants but inadequate in some of their mothers and in formula‐fed infants. Journal of Pediatric Gastroenterology and Nutrition, 5(2), 283–289. 3958855

[ajpa24603-bib-0072] Selhub, J. , Arnold, R. , Smith, A. M. , & Picciano, M. F. (1984). Milk folate binding‐protein (FBP)—A secretory protein for folate. Nutrition Research, 4(2), 181–187. 10.1016/s0271-5317(84)80003-2

[ajpa24603-bib-0073] Shell‐Duncan, B. , & Yung, S. A. (2004). The maternal depletion transition in northern Kenya: The effects of settlement, development and disparity. Social Science & Medicine, 58(12), 2485–2498. 10.1016/j.socscimed.2003.09.016 15081199

[ajpa24603-bib-0074] Stabler, S. P. (2010). Clinical folate deficiency. In Lynn B Bailey (Ed.), Folate in health and disease (2nd ed., pp. 409–428). CRC Press, Taylor and Francis Group.

[ajpa24603-bib-0075] Stearns, S. C. (1992). The evolution of life histories. Oxford: Oxford University Press.

[ajpa24603-bib-0076] Stinson, S. (1985). Sex differences in environmental sensitivity during growth and development. American Journal of Physical Anthropology, 28(S6), 123–147.

[ajpa24603-bib-0077] Tamura, T. , & Picciano, M. F. (2006). Folate and human reproduction. American Journal of Clinical Nutrition, 83(5), 993–1016. 16685040 10.1093/ajcn/83.5.993

[ajpa24603-bib-0078] Tamura, T. , Picciano, M. F. , & McGuire, M. K. (2009). Folate in pregnancy and lactation. CRC Press.

[ajpa24603-bib-0079] Tin, T. , & Fujita, M. (2020). Investment in innate immune defense in northern Kenya. American Journal of Physical Anthropology, 171, 287.

[ajpa24603-bib-0080] Trugo, N. M. F. , Donangelo, C. M. , Koury, J. C. , Barreto Silva, M. I. , & Freitas, L. A. (1988). Concentration and distribution pattern of selected micronutrients in preterm and term milk from urban Brazilian mothers during early lactation. European Journal of Clinical Nutrition, 42(6), 497–507. 3409858

[ajpa24603-bib-0081] UCLA Statistical Consulting Group . (2021). Introduction to power analysis. UCLA.

[ajpa24603-bib-0082] Udipi, S. A. , Kirksey, A. , & Roepke, J. L. B. (1987). Diurnal‐variations in folacin levels of human‐milk—Use of a single sample to represent folacin concentration in milk during a 24‐h period. American Journal of Clinical Nutrition, 45(4), 770–779. 10.1093/ajcn/45.4.770 3565305

[ajpa24603-bib-0083] USAID . (2018). Economic resilience to drought: Kenya analysis. https://www.usaid.gov/sites/default/files/documents/1867/Kenya_Economics_of_Resilience_Final_Jan_4_2018_-_BRANDED.pdf

[ajpa24603-bib-0084] Villalpando, S. , & del Prado, M. (1999). Interrelation among dietary energy and fat intakes, maternal body fatness, and milk total lipid in humans. Journal of Mammary Gland Biology and Neoplasia, 4(3), 285–295. 10.1023/a:1018702030259 10527470

[ajpa24603-bib-0085] Villalpando, S. , Latulippe, M. E. , Rosas, G. , Irurita, M. J. , Picciano, M. F. , & O'Connor, D. L. (2003). Milk folate but not milk iron concentrations may be inadequate for some infants in a rural farming community in San Mateo, Capulhuac, Mexico. American Journal of Clinical Nutrition, 78(4), 782–789. 14522737 10.1093/ajcn/78.4.782

[ajpa24603-bib-0086] Wander, K. , Shell‐Duncan, B. , & McDade, T. W. (2009). Evaluation of iron deficiency as a nutritional adaptation to infectious disease: An evolutionary medicine perspective. American Journal of Human Biology, 21(2), 172–179. 10.1002/ajhb.20839 18949769 PMC3938201

[ajpa24603-bib-0087] Wasserstein, R. L. , Schirm, A. L. , & Lazar, N. A. (2019). Moving to a world beyond “p < 0.05”. American Statistician, 73, 1–19. 10.1080/00031305.2019.1583913

[ajpa24603-bib-0088] World Health Organization . (2006). Kenya: Health action in drought affected districts. Weekly Bulletin, 15th October 2006

[ajpa24603-bib-0089] World Health Organization . (2017). Nutritional anaemias: Tools for effective prevention and control (p. 83). World Health Organization.

