# Supplementary material for: Characterizing the extent human milk folate is buffered against maternal malnutrition and infection in drought‐stricken northern Kenya
Source: Am J Biol Anthropol. 2022 Aug 23;179(2):171–83. doi: 10.1002/ajpa.24603 (PMC9805107; doi:10.1002/ajpa.24603)
Supplement: Supplementary file 1 — TABLE S1 Regression models for milk folate receptor‐α (FOLR1, log‐transformed) with an interaction term for infant age having >0.1 probability, using lower (A) and higher (B) hyperhomocysteinemia cutoffs. [file AJPA-179-171-s002.docx]

Table S1 Regression models for milk folate receptor-α (FOLR1, log-transformed) with an interaction term for infant age having > 0.1 probability, using lower (*A*) and higher (*B*) hyperhomocysteinemia cutoffs.

*A*. Outcome Milk FOLR1 (ln) n=203

|  | Model 4a | | | | Model 5a | | | | Model 6a | | | |
| --- | --- | --- | --- | --- | --- | --- | --- | --- | --- | --- | --- | --- |
| Predictors | Coef. | β | SE | *P* | Coef. | β | SE | *P* | Coef. | β | SE | *P* |
| Underweight | 0.059 | 0.090 | 0.036 | 0.105 | 0.066 | 0.101 | 0.036 | 0.070 | 0.063 | 0.096 | 0.036 | 0.084 |
| Iron deficiency anemia | -0.002 | -0.003 | 0.046 | 0.959 | -0.018 | -0.022 | 0.045 | 0.692 | -0.016 | -0.020 | 0.045 | 0.723 |
| HHcy ^a^ | 0.120 | 0.135 | 0.049 | 0.015 | 0.107 | 0.120 | 0.051 | 0.037 | 0.119 | 0.134 | 0.049 | 0.016 |
| Inflammation | 0.034 | 0.042 | 0.044 | 0.447 | 0.029 | 0.036 | 0.044 | 0.513 | 0.028 | 0.035 | 0.045 | 0.524 |
| Age | -0.027 | -0.389 | 0.004 | 0.000 | -0.023 | -0.333 | 0.004 | 0.000 | -0.024 | -0.355 | 0.004 | 0.000 |
| Sex (male) | -0.045 | -0.073 | 0.034 | 0.187 | -0.051 | -0.083 | 0.034 | 0.131 | -0.050 | -0.082 | 0.034 | 0.140 |
| Iron deficiency anemia × Age | 0.015 | 0.097 | 0.010 | 0.124 |  |  |  |  |  |  |  |  |
| HHcy × Age |  |  |  |  | -0.011 | -0.056 | 0.012 | 0.352 |  |  |  |  |
| Inflammation × Age |  |  |  |  |  |  |  |  | 0.002 | 0.010 | 0.010 | 0.863 |
| Milk total protein (ln) | 0.717 | 0.441 | 0.094 | 0.000 | 0.714 | 0.439 | 0.094 | 0.000 | 0.712 | 0.438 | 0.095 | 0.000 |
| Constant | 6.372 | . | 0.031 | 0.000 | 6.374 | . | 0.031 | 0.000 | 6.374 | . | 0.031 | 0.000 |
| Model *P* | 0.000 |  |  |  | 0.000 |  |  |  | 0.000 |  |  |  |
| R^2^ | 0.430 |  |  |  | 0.425 |  |  |  | 0.423 |  |  |  |
| Adjusted R^2^ | 0.406 |  |  |  | 0.401 |  |  |  | 0.399 |  |  |  |
| Mean VIF | 1.12 |  |  |  | 1.10 |  |  |  | 1.10 |  |  |  |

^a^ HHcy, hyperhomocysteinemia (serum homocysteine > 12 µmol/l); VIF, variance inflation factor

*B*. Outcome Milk FOLR1 (ln) n=203

|  | Model 4b | | | | Model 5b | | | | Model 6b | | | |
| --- | --- | --- | --- | --- | --- | --- | --- | --- | --- | --- | --- | --- |
| Predictors | Coef. | β | SE | *P* | Coef. | β | SE | *P* | Coef. | β | SE | *P* |
| Underweight | 0.056 | 0.086 | 0.036 | 0.120 | 0.062 | 0.095 | 0.037 | 0.090 | 0.061 | 0.093 | 0.036 | 0.094 |
| Iron deficiency anemia | -0.011 | -0.014 | 0.046 | 0.812 | -0.026 | -0.032 | 0.045 | 0.565 | -0.025 | -0.031 | 0.045 | 0.578 |
| HHcy ^b^ | 0.138 | 0.122 | 0.062 | 0.028 | 0.129 | 0.113 | 0.066 | 0.054 | 0.133 | 0.117 | 0.063 | 0.035 |
| Inflammation | 0.029 | 0.035 | 0.045 | 0.520 | 0.024 | 0.030 | 0.045 | 0.594 | 0.023 | 0.029 | 0.045 | 0.602 |
| Age | -0.027 | -0.397 | 0.004 | 0.000 | -0.024 | -0.355 | 0.004 | 0.000 | -0.025 | -0.360 | 0.004 | 0.000 |
| Sex (male) | -0.046 | -0.074 | 0.034 | 0.180 | -0.052 | -0.084 | 0.034 | 0.131 | -0.052 | -0.084 | 0.034 | 0.131 |
| Iron deficiency anemia × Age | 0.016 | 0.101 | 0.010 | 0.110 |  |  |  |  |  |  |  |  |
| HHcy × Age |  |  |  |  | -0.003 | -0.012 | 0.017 | 0.842 |  |  |  |  |
| Inflammation × Age |  |  |  |  |  |  |  |  | 0.001 | 0.008 | 0.010 | 0.900 |
| Milk total protein (ln) | 0.715 | 0.440 | 0.094 | 0.000 | 0.708 | 0.435 | 0.095 | 0.000 | 0.709 | 0.436 | 0.095 | 0.000 |
| Constant | 6.382 | . | 0.030 | 0.000 | 6.383 | . | 0.030 | 0.000 | 6.384 | . | 0.030 | 0.000 |
| Model *P* | 0.000 |  |  |  | 0.000 |  |  |  | 0.000 |  |  |  |
| R^2^ | 0.426 |  |  |  | 0.419 |  |  |  | 0.419 |  |  |  |
| Adjusted R^2^ | 0.402 |  |  |  | 0.395 |  |  |  | 0.395 |  |  |  |
| Mean VIF | 1.12 |  |  |  | 1.10 |  |  |  | 1.10 |  |  |  |

^b^ HHcy, hyperhomocysteinemia (serum homocysteine > 14 µmol/l); VIF, variance inflation factor
