# Supplementary material for: Characterizing the extent human milk folate is buffered against maternal malnutrition and infection in drought‐stricken northern Kenya
Source: Am J Biol Anthropol. 2022 Aug 23;179(2):171–83. doi: 10.1002/ajpa.24603 (PMC9805107; doi:10.1002/ajpa.24603)
Supplement: Supplementary file 2 — TABLE S2 Regression models for milk folate receptor‐α (FOLR1, log‐transformed) with an interaction term for infant sex having >0.1 probability, using lower (A) and higher (B) hyperhomocysteinemia cutoffs. [file AJPA-179-171-s001.docx]

Table S2 Regression models for milk folate receptor-α (FOLR1, log-transformed) with an interaction term for infant sex having > 0.1 probability, using lower (*A*) and higher (*B*) hyperhomocysteinemia cutoffs.

*A*. Outcome Milk FOLR1 (ln) n=203

|  | Model 7a | | | | Model 8a | | | | Model 9a | | | |
| --- | --- | --- | --- | --- | --- | --- | --- | --- | --- | --- | --- | --- |
| Predictors | Coef. | β | SE | *P* | Coef. | β | SE | *P* | Coef. | β | SE | *P* |
| Underweight | 0.029 | 0.044 | 0.052 | 0.585 | 0.062 | 0.096 | 0.036 | 0.085 | 0.067 | 0.103 | 0.036 | 0.067 |
| Iron deficiency anemia | -0.014 | -0.017 | 0.045 | 0.756 | 0.013 | 0.016 | 0.068 | 0.847 | -0.019 | -0.023 | 0.045 | 0.677 |
| HHcy ^a^ | 0.121 | 0.136 | 0.049 | 0.015 | 0.119 | 0.134 | 0.049 | 0.016 | 0.114 | 0.129 | 0.049 | 0.022 |
| Inflammation | 0.033 | 0.041 | 0.045 | 0.456 | 0.031 | 0.038 | 0.045 | 0.493 | -0.015 | -0.018 | 0.069 | 0.829 |
| Age | -0.024 | -0.355 | 0.004 | 0.000 | -0.024 | -0.355 | 0.004 | 0.000 | -0.024 | -0.350 | 0.004 | 0.000 |
| Sex (male) | -0.073 | -0.118 | 0.042 | 0.081 | -0.042 | -0.068 | 0.037 | 0.262 | -0.063 | -0.103 | 0.037 | 0.089 |
| Underweight × Sex | 0.066 | 0.079 | 0.073 | 0.361 |  |  |  |  |  |  |  |  |
| Iron deficiency anemia × Sex |  |  |  |  | -0.052 | -0.051 | 0.089 | 0.565 |  |  |  |  |
| Inflammation × Sex |  |  |  |  |  |  |  |  | 0.076 | 0.074 | 0.091 | 0.405 |
| Milk total protein (ln) | 0.702 | 0.431 | 0.095 | 0.000 | 0.712 | 0.438 | 0.094 | 0.000 | 0.707 | 0.434 | 0.094 | 0.000 |
| Constant | 6.386 | . | 0.033 | 0.000 | 6.369 | . | 0.032 | 0.000 | 6.381 | . | 0.032 | 0.000 |
| Model *P* | 0.000 |  |  |  | 0.000 |  |  |  | 0.000 |  |  |  |
| R^2^ | 0.425 |  |  |  | 0.423 |  |  |  | 0.425 |  |  |  |
| Adjusted R^2^ | 0.401 |  |  |  | 0.400 |  |  |  | 0.401 |  |  |  |
| Mean VIF | 1.45 |  |  |  | 1.46 |  |  |  | 1.46 |  |  |  |

^a^ HHcy, hyperhomocysteinemia (serum homocysteine > 12 µmol/l); VIF, variance inflation factor

*B*. Outcome Milk FOLR1 (ln) n=203

|  | Model 7b | | | | Model 8b | | | | Model 9b | | | |
| --- | --- | --- | --- | --- | --- | --- | --- | --- | --- | --- | --- | --- |
| Predictors | Coef. | β | SE | *P* | Coef. | β | SE | *P* | Coef. | β | SE | *P* |
| Underweight | 0.035 | 0.053 | 0.052 | 0.511 | 0.061 | 0.093 | 0.036 | 0.096 | 0.066 | 0.100 | 0.036 | 0.074 |
| Iron deficiency anemia | -0.024 | -0.029 | 0.045 | 0.601 | 0.004 | 0.005 | 0.068 | 0.954 | -0.028 | -0.034 | 0.045 | 0.540 |
| HHcy ^b^ | 0.131 | 0.115 | 0.063 | 0.038 | 0.134 | 0.118 | 0.063 | 0.034 | 0.128 | 0.113 | 0.063 | 0.043 |
| Inflammation | 0.027 | 0.034 | 0.045 | 0.545 | 0.025 | 0.031 | 0.045 | 0.571 | -0.024 | -0.029 | 0.069 | 0.728 |
| Age | -0.025 | -0.361 | 0.004 | 0.000 | -0.025 | -0.361 | 0.004 | 0.000 | -0.024 | -0.355 | 0.004 | 0.000 |
| Sex (male) | -0.069 | -0.112 | 0.042 | 0.099 | -0.043 | -0.070 | 0.037 | 0.250 | -0.066 | -0.107 | 0.037 | 0.078 |
| Underweight × Sex | 0.052 | 0.061 | 0.073 | 0.481 |  |  |  |  |  |  |  |  |
| Iron deficiency anemia × Sex |  |  |  |  | -0.051 | -0.051 | 0.090 | 0.568 |  |  |  |  |
| Inflammation × Sex |  |  |  |  |  |  |  |  | 0.083 | 0.081 | 0.091 | 0.363 |
| Milk total protein (ln) | 0.701 | 0.431 | 0.095 | 0.000 | 0.710 | 0.436 | 0.095 | 0.000 | 0.704 | 0.432 | 0.095 | 0.000 |
| Constant | 6.393 | . | 0.033 | 0.000 | 6.379 | . | 0.032 | 0.000 | 6.391 | . | 0.031 | 0.000 |
| Model *P* | 0.000 |  |  |  | 0.000 |  |  |  | 0.000 |  |  |  |
| R^2^ | 0.420 |  |  |  | 0.420 |  |  |  | 0.421 |  |  |  |
| Adjusted R^2^ | 0.396 |  |  |  | 0.396 |  |  |  | 0.397 |  |  |  |
| Mean VIF | 1.45 |  |  |  | 1.45 |  |  |  | 1.45 |  |  |  |

^b^ HHcy, hyperhomocysteinemia (serum homocysteine > 14 µmol/l); VIF, variance inflation factor
